# Comparison of therapeutic efficacy in depression between repetitive TMS and deep TMS

**DOI:** 10.1007/s00702-025-02944-w

**Published:** 2025-05-27

**Authors:** Walter Paganin, Lorenzo Maria Contini

**Affiliations:** 1 University of Rome Tor Vergata, Rome, EU Italy; 2 University of Rome, Tor Vergata Via Cracovia 50, 00133 Rome, EU Italy

**Keywords:** Transcranial magnetic stimulation, TMS, rTMS, dTMS, Depression, TRD, DTD

## Abstract

Transcranial Magnetic Stimulation (TMS) is a non-invasive neuromodulation technique with promising potential in treating psychiatric and neurological disorders. By offering an alternative to surgery or medication, TMS could significantly enhance patient outcomes and quality of life, particularly for those with conditions resistant to traditional therapies. With ongoing research advancements, TMS may become a cornerstone of personalized medicine, enabling treatments tailored to individual brain patterns and needs. Although rTMS has FDA approval and dTMS has clearance for treating treatment-resistant depression (TRD), comparative studies between these techniques remain scarce. This systematic review aims to assess and compare the efficacy of rTMS and dTMS in TRD through the analysis of observational studies and clinical trials that directly compare these two treatments. Relevant literature was identified through searches in Web of Science, Scopus, Cochrane Library, PsycINFO, PubMed, and Open Gray, covering studies published in English from 2014 to 2024. Only three studies, including one observational study and two RCTs, met inclusion criteria, indicating similar efficacy and safety for both techniques. This review highlights that both rTMS and dTMS are effective and safe for treating TRD, with no significant differences in symptom remission. However, dTMS is significantly more expensive, making rTMS a more viable option in resource-limited settings. The review underscores the need for further multicenter and long-term studies to better clarify the cost-effectiveness of these treatments.

**Supplementary Information:**

The online version contains supplementary material available at 10.1007/s00702-025-02944-w.

## Introdution

Magnetism has traversed the boundaries between science, pseudoscience, and alternative healing, marking a fascinating path in human history that has influenced medical and philosophical thought, particularly in the 18th and 19th centuries. Magnetism and electromagnetism, phenomena we now understand through physics, were long considered mystical and enigmatic forces, with ancient roots in natural observations and the use of magnets. Since antiquity, magnets have been attributed with healing properties. The term “magnet” itself derives from the ancient city of Magnesia in Asia Minor (modern-day Manisa, Turkey), where magnetic stones were first discovered around 800 BCE. Thales of Miletus, who lived nearby, was likely the first Greek to study their effects (Robinson F. Neville H et al., no date). Greek physicians of the time believed magnets could “extract” disease from the human body, having observed that magnetic fields could attract iron even through materials such as wood. In the temples of Asclepius, from the 5th to 4th century BCE, healers known as “somniatores” employed magnetism and sleep to identify the causes of diseases and prescribe remedies (Kellie 1999). Hippocrates of Cos, who lived between 460 and 370 BCE, also used magnets for therapeutic purposes. Early theories on magnetism were largely speculative, based on empirical observations. However, with the scientific advancements of the 17th and 18th centuries, magnetism began to be studied more systematically. Mesmerism, on the other hand, originated from the work of German physician Franz Anton Mesmer (1734–1815), who proposed the existence of an invisible “animal magnetism” pervading the universe and the human body. According to Mesmer, physical and mental ailments were due to blockages of this fluid, which could be influenced and rebalanced using magnets and specific hand movements over the patient’s body. Mesmer initially practiced animal magnetism in Vienna during the 1770s, employing magnets in his treatments, though he later concluded that the magnets themselves were unnecessary, as he believed his own body radiated a magnetic field transferable to the patient. Despite its initial popularity, mesmerism was met with skepticism by the medical community. In 1784, under the directive of King Louis XVI, a scientific commission concluded that there was no evidence for the existence of “animal magnetism,” attributing observed effects to suggestion. This verdict greatly undermined mesmerism’s scientific credibility. However, the phenomenon was later examined as a psychological process by British physician James Braid (1785–1860), who reinterpreted mesmerism physiologically. Braid coined the terms “hypnosis” and “neuro-hypnotism,” understanding that Mesmer’s effects were not due to magnetic fluids but to induced states of suggestion and mental concentration (Paganin and Signorini 2021). In the 19th century, a significant advancement in bioelectromagnetic understanding came with the development of neurophysiology. Pioneering studies, such as those by Luigi Galvani (1737–1798) and Emil du Bois-Reymond (1818–1896), explored biological electricity, demonstrating that nerves and muscles functioned through electrical impulses (Hess 1994). In 1870, German physicians Gustav Fritsch and Eduard Hitzig investigated the effects of electrical stimulation on the brain. By applying electrical stimuli to the motor cortex of dogs, they discovered that specific cortical areas controlled precise, contralateral muscular movements. This groundbreaking work provided the first evidence of brain localization of motor functions, establishing that distinct brain areas govern specific movements (Hagner 2012). By the early 20th century, therapeutic brain stimulation techniques began to emerge. Electroconvulsive therapy (ECT), introduced in the 1930s, used electrical currents to induce controlled seizures in patients with severe mental disorders. This technique was pioneered by the Italian neuropsychiatrist Ugo Cerletti (1877–1963), who, along with his collaborator Lucio Bini (1908–1964), developed ECT in 1938 as a treatment for conditions like schizophrenia and major depression. In the 1950s and 1960s, advancements in cerebral electrophysiology led researchers like Spanish neurophysiologist José Manuel Rodriguez Delgado (1915–2011) to explore electrical stimulation’s effects on human behavior. Delgado conducted pioneering experiments by implanting electrodes into the brains of animals and, in certain instances, humans, to observe how electrical stimulation could modulate emotions and behaviors. (Delgado et al. 1952). Although he did not use magnetic fields, Delgado pioneered the possibility of influencing brain activity through electromagnetic energy. Similarly, in the 1950s, German neurosurgeons Wilder Penfield (1891–1976) and Herbert Jasper (1906–1999) conducted in-depth studies on direct cortical stimulation during neurosurgical procedures on epileptic patients. Collaborating with Theodore Rasmussen (1910–2002), Penfield developed a detailed map of the homunculus motorio, a somatotopic representation of the motor cortex that illustrates how specific cortical regions are dedicated to controlling precise parts of the body (Stahnisch and Nakashima 2013). These studies laid the foundation for modern electrophysiology and influenced the development of technologies to stimulate the brain through electric and magnetic fields. In the 1970s, although primarily focused on the therapeutic use of electric fields, various researchers continued exploring the potential of electrical and magnetic neuromodulation. However, TMS was not systematically developed until 1985.

The first TMS device was developed in 1985 by Anthony Barker (born in 1950) and his colleagues at the University of Sheffield. Using pulsed magnetic fields, TMS induces electric currents in specific areas of the brain without direct contact, allowing for non-invasive stimulation of the motor cortex and other brain regions. Transcranial Magnetic Stimulation provides a targeted approach to stimulating specific brain areas without the side effects associated with ECT (Barker et al. 1985a). The journey of TMS, which began in 1985 as a groundbreaking discovery, marked the birth of this transformative technology. This initial step laid the foundation for future advancements, opening new possibilities in brain research. Between the late 1980s and early 1990s, researchers began exploring TMS’s potential as a tool for investigating brain function and connectivity. During this period, studies using TMS to map brain areas proliferated, aiming to understand their roles in various cognitive and motor processes (George et al. 2002). Researchers found that TMS can temporarily suppress or enhance brain activity in specific regions, thus providing valuable insights into brain organization and function. The 1990s marked a significant advancement in TMS technology with the introduction of repetitive TMS (rTMS), allowing for prolonged effects on brain activity that extended beyond the duration of each application. This development opened new avenues for research and therapeutic use by enabling sustained modulation of brain activity (Daskalakis et al. 2002). In 1995, researchers began exploring TMS as a potential treatment for depression, initiating a transformative era in psychiatric therapies that offered hope for patients unresponsive to conventional treatments. Early promising results led to further studies and the establishment of TMS protocols for depression treatment (Jannati et al. 2023).

A major milestone was reached in 2008 when the U.S. Food and Drug Administration (FDA) approved TMS for major depressive disorder in adults resistant to antidepressant medications (Perera et al. 2016). This approval marked TMS’s transition from an experimental method to a recognized medical treatment, substantially expanding its clinical applications (Karris et al. 2017). The 2010s witnessed further expansion in TMS research, as scientists and clinicians investigated its potential for treating various neurological and psychiatric conditions, including anxiety disorders, chronic pain, and post-stroke rehabilitation, thereby demonstrating TMS’s versatility in addressing a broad spectrum of brain disorders. In 2013, another technological leap occurred with the introduction of deep TMS (dTMS), which enabled stimulation of previously inaccessible deeper brain regions (Rapinesi et al. 2015). This advancement broadened TMS’s application scope, allowing researchers to target brain structures involved in complex neurological and psychiatric conditions (Xin et al. 2021), such as difficult-to-treat depression (DTD) a distinct clinical construct beyond TRD.

While TRD is typically defined by an inadequate response to at least two antidepressant trials of adequate dose and duration, DTD encompasses a multidimensional framework in which psychosocial, biological, and systemic factors (e.g., psychiatric or medical comorbidities, childhood trauma, adherence issues) hinder the achievement of full symptom control. Patients with DTD often present with persistent symptoms such as anhedonia, anxiety, and significant functional impairment, despite receiving evidence-based care. DTD reflects a therapeutic continuum ranging from partial response to complete nonresponse, thereby shifting the clinical focus from full remission to optimizing symptom management. Its management requires integrated, multidisciplinary strategies, including pharmacological treatment, psychotherapy, neuromodulation techniques (such as TMS/dTMS), social interventions, and active involvement of both the patient and their family (Paganin 2023). This was followed by incremental FDA clearances for dTMS, initially for major depressive disorder in 2013, specifically targeting treatment-resistant depression (Levkovitz et al. 2015). “FDA Approved” indicates an authorization process that requires a rigorous examination of safety and effectiveness; “FDA Cleared” applies to low- or medium-risk medical devices that demonstrate substantial equivalence to an already marketed device; and “CE certification” indicates compliance with the safety and health standards required in the European Economic Area, which was granted in Europe for both types of technology. In 2018, the FDA granted dTMS clearance for obsessive-compulsive disorder (OCD), strengthening the role of TMS in psychiatric treatment and expanding options for patients with this challenging condition (Brakoulias et al. 2021); (Maia et al. 2022). In 2020, dTMS also received FDA clearance for smoking cessation, demonstrating its efficacy in reducing cravings and withdrawal symptoms in adult smokers and in 2021, dTMS received FDA clearance to treat anxious depression as a distinct indication from TRD.

This systematic review aims to elucidate the comparative efficacy of rTMS and dTMS in patients with TRD. By analyzing observational studies and clinical trials that assess the techniques, protocols, side effects, and cost implications of rTMS and dTMS, the findings intend to inform clinical decision-making, optimize treatment protocols, and advance non-invasive brain stimulation therapies in the management of depression.

## Materials and methods

### Search strategy

The search was conducted using the following databases: Web of Science, Scopus, Cochrane Library, PsycINFO, PubMed, Open Gray, and trial registries such as ClinicalTrials.gov, with the following search strings:

**Web of Science**:

(“Deep Transcranial Magnetic Stimulation” OR “dTMS”) AND (“Transcranial Magnetic Stimulation” OR “TMS”) AND (“Major Depressive Disorder”), with 35 documents identified.

**Scopus**:

(TITLE-ABS-KEY (“Deep Transcranial Magnetic Stimulation” OR dtms) AND TITLE-ABS-KEY (“Transcranial Magnetic Stimulation” OR tms) AND TITLE-ABS-KEY (“Major Depressive Disorder” OR “Treatment-Resistant Depression” OR “Difficult to Treat Depression” OR “resistant depression” OR “refractory depression”)) AND (LIMIT-TO (DOCTYPE, “ar”)) AND (LIMIT-TO (PUBYEAR, 2014) OR LIMIT-TO (PUBYEAR, 2015) OR LIMIT-TO (PUBYEAR, 2016) OR LIMIT-TO (PUBYEAR, 2017) OR LIMIT-TO (PUBYEAR, 2018) OR LIMIT-TO (PUBYEAR, 2019) OR LIMIT-TO (PUBYEAR, 2020) OR LIMIT-TO (PUBYEAR, 2021) OR LIMIT-TO (PUBYEAR, 2022) OR LIMIT-TO (PUBYEAR, 2023) OR LIMIT-TO (PUBYEAR, 2024)) AND (LIMIT-TO (LANGUAGE, “English”) OR LIMIT-TO (LANGUAGE, “Italian”) OR LIMIT-TO (LANGUAGE, “French”) OR LIMIT-TO (LANGUAGE, “Spanish”)), with 34 documents identified.

**Cochrane Library**:

((depressive disorder, treatment-resistant), ab, kw AND (“transcranial magnetic stimulation”),ab, kw AND (deep transcranial magnetic stimulation), ab, kw NOT (older), ab, kw NOT (pediatrics), ab, kw). with 27 documents identified.

**PsycINFO**:

(DeepTMS OR Any Field: dTMS AND Any Field: Depression AND Year: 2014 To 2024), with 44 documents identified.

**PubMed**:

((TRANSCRANIAL MAGNETIC STIMULATION) AND (DEEP TRANSCRANIAL MAGNETIC STIMULATION)) AND (DEPRESSIVE DISORDER, TREATMENT-RESISTANT), with 9 documents identified.

**Open Gray**:

(Deep TMS and Standard TMS in Major Depression), with 0 documents identified.

**ClinicalTrials.gov**:

(Deep TMS vs. Standard TMS), 2 ongoing trials, not included in this review.

Effects of a Classic High-frequency rTMS Treatment Versus a Deep rTMS Treatment (HAUVERDEEP).

ClinicalTrials.gov / ID NCT04956016.

*Centre Hospitalier Henri Laborit*, *France*, *152 patients*, *study completion: end of 2025.*

*Primary objective: demonstrate that dTMS is more effective than high-frequency rTMS with a conventional coil.*

*Arm A: standard treatment with rTMS (using figure-of-8 coil) and standard therapy.*

*Arm B: treatment with deep TMS (using H-coil*, *“helmet”) and standard therapies.*

*Protocol includes 20 rTMS sessions (5 sessions per week) and 3 follow-up visits: Day 30*, *Day 60*, *and Day 90.*

*Repetitive Versus Deep Transcranial Magnetic Stimulation for Major Depression (ReDeeMD)*.

ClinicalTrials.gov / ID NCT05902312.

*Centre hospitalier de l’Université de Montréal*, *Canada*, *50 patients*, *study completion: September 2027.*

*This randomized controlled trial aims to assess the efficacy of two different TMS techniques in TRD: repetitive TMS (rTMS) and deep TMS (dTMS).*

*Population: patients with major depressive disorder.*

*Objectives: to evaluate the superiority of dTMS over rTMS in TRD and the predictive capacity of scalable candidate biomarkers.*

*Participants will be randomly assigned to intervention groups (rTMS or dTMS). dTMS provides a broader magnetic field than rTMS*, *potentially reducing coil misplacement errors and improving cortical stimulation accuracy.*

**Fig. 1:**
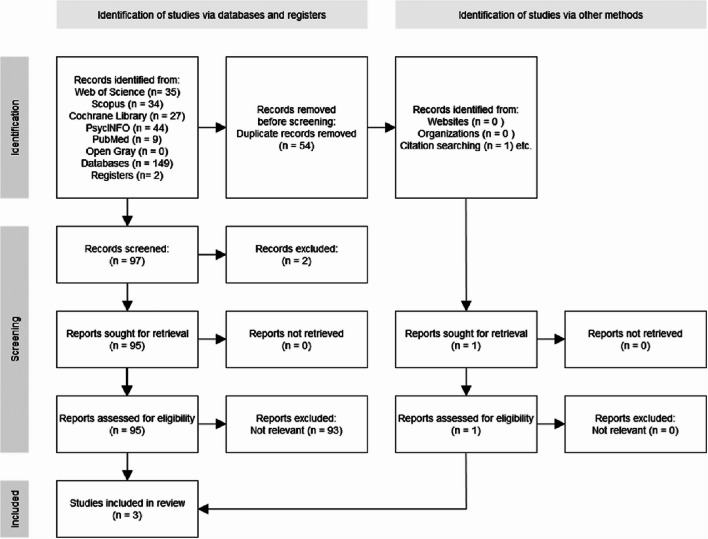
PRISMA Flow diagram for systematic literature review

Additional search methods included citation tracking, consultation of specialist websites, and scientific organizations. Keywords used included combinations of “Deep Transcranial Magnetic Stimulation,” “dTMS,” “Transcranial Magnetic Stimulation,” “TMS,” “Major Depressive Disorder,” “Treatment-Resistant Depression,” and “Difficult Depression Treatment” (DTD). The search was restricted to studies published within the last 10 years, including observational real word studies and randomized controlled trials (RCTs) that directly compare dTMS and rTMS in adult patients with TRD/DTD, excluding reviews, case reports, non-randomized studies, and studies conducted on pediatric populations. All documents were selected to cover a 10-year span (2014–2024, from January 1, 2014, to July 31, 2024), to include the most up-to-date and relevant research. Studies were sought from a variety of clinical and geographical contexts to ensure greater data representativeness (see Fig. 1).

### Study selection

Publications retrieved from the databases were initially screened at the title and abstract level by two independent, blinded reviewers (WP, LC). Discrepancies were resolved through discussion and consensus. Reports identified as potentially relevant were then fully evaluated based on explicit selection criteria. Inclusion criteria comprised RCTs comparing rTMS and dTMS in terms of clinical improvement in depressive symptoms, measured by standardized scales, as well as assessments of any side effects. Observational real studies were included as well. Studies that did not report these outcomes were excluded from the analysis.

### Data extraction and quality assessment

Data were extracted from the included studies following a standardized protocol, collecting details on participant characteristics (age, sex, depression duration), intervention specifics (treatment frequency and duration), and outcomes (symptom improvement, remission rates, side effects). Study quality was independently assessed by two blinded reviewers (WP, LC) using STROBE guidelines for the observational study and the Jadad Scale and Cochrane Risk of Bias Checklist for RCTs studies (see Table 1).

The STROBE guidelines provide criteria for clarity and reliability to ensure transparent reporting of observational studies. They include specifying the Title and Abstract, which should describe the study design and summarize methods, results, and conclusions; the Introduction, covering the study rationale and objectives; Methods, detailing study design, population, variables, data collection, and statistical analysis, including confounder control and handling of missing data; Results, presenting complete data with statistical analysis and confidence intervals; the Discussion, interpreting results along with limitations, biases, and generalizability; and finally, Conclusions, addressing clinical implications and directions for future research (Von Elm et al. 2007).

The Jadad Scale, developed by Alejandro Jadad in 1996, assesses the quality of RCTs based on three criteria: randomization, blinding, and handling of follow-up losses. Each criterion, if well met, adds one point to a maximum score of 5, with higher scores reflecting better quality. This scale is widely used in systematic reviews and meta-analyses to assess RCT quality and minimize bias (Jadad et al. 1996). The Cochrane Risk of Bias Checklist, developed by the Cochrane Collaboration (Higgins et al. 2011), is a systematic tool for assessing potential biases in RCTs. This checklist categorizes bias risk as “low,” “high,” or “uncertain” across key areas:

- Selection Bias: Evaluates whether participants were appropriately randomized and if group allocation was concealed (allocation concealment) to prevent researcher influence on assignment.
- Performance Bias: Assesses the blinding of participants and personnel during the intervention, ensuring equal treatment regardless of group assignment.
- Attrition Bias: Analyzes how losses to follow-up were managed, checking if losses were balanced across groups and if missing data were handled to prevent influencing outcomes.
- Detection Bias: Assesses if outcome assessors were blinded to group assignments to minimize the risk of their knowledge impacting measured results.
- Reporting Bias: Examines whether all study results, including expected and relevant outcomes, were reported accurately to avoid selective reporting of favorable outcomes.
- Other Sources of Bias: Includes any additional sources of bias, such as conflicts of interest or funding by industry sources.

This structured framework enables a comprehensive evaluation of RCT quality and helps mitigate the impact of bias on study findings.

### Results synthesis

The extracted data were analyzed and thematically synthesized to identify common patterns and differences among the various studies. The thematic synthesis allowed for grouping studies based on their primary objectives and outcome measurement methods. In the presence of significant heterogeneity, a narrative synthesis of the results was provided, highlighting the strengths and limitations of individual studies (see Tables 1 and 2).

**Table 1:** Overview of study characteristics

Study Title	Authors	Jadad Scale (Score)	STROBE Assessment	Cochrane Checklist (Bias Categories)	Objective
A Comparison of rTMS and dTMS Treatment Outcomes for Major Depressive Disorder in a Real-World Clinical Practice Setting.	Theodore Wirecki et al., 2015	Observational	The study has strengths, including a clear objective and use of the validated Beck Depression Inventory scale for outcomes. However, it lacks sufficient detail on key aspects like sample selection and confounder control.	High risk of selection bias due to lack of randomization and potential confounding bias from unaddressed confounders.	This study aims to compare the efficacy of rTMS and dTMS devices for the treatment of MDD in a real-world clinical setting. The goal is to provide practical data on the effectiveness of each device to better inform clinical practice. This study carries a high overall risk of bias, particularly due to the lack of randomization, blinding, and control of confounding variables.
Efficacy of repetitive transcranial magnetic stimulation using an H1-Coil or figure-8-Coil in the treatment of unipolar major depressive disorder	Igor Filipčić et al. 2018	Randomized RCT including a control group. However, blinding is partial (single-blinded), with no true blinding for patients. Estimated Jadad score: 3/5.		It has limitations due to performance bias and potential attrition bias stemming from the lack of patient blinding.	The study is designed as a randomized controlled clinical trial aimed at evaluating differences in efficacy and safety between TMS treatments using the H1 coil and the figure-8 coil in patients with MDD. The primary objective is to assess the short-term efficacy of these treatments. The secondary objective is to examine biomarkers such as Brain-Derived Neurotrophic Factor (BDNF) with a focus on remission of specific target symptoms, including sleep and hope measured by MADRS, and Pittsburgh Sleep Quality Index.
Efficacy and Safety of Repetitive Transcranial Magnetic Stimulation Using an H1-Coil or Figure-8-Coil in the Treatment of Unipolar Major Depressive Disorder	Igor Filipčić et al. 2019	RCT with a rigorous design, including details on randomization, partial blinding, and the use of a control group. Estimated Jadad score: 4/5, as it lacks full double blinding.		Selection bias is minimized due to randomization; however, there is a risk of performance bias as the treatment was not fully blinded. Low risk in all other bias categories.	The aim of this study is to evaluate and compare the clinical outcomes of two FDA-approved TMS protocols using the H1 coil and the figure-8 coil as adjunctive treatments for treatment-resistant MDD. The study focuses on the proportion of patients achieving remission of depressive symptoms, analyzing the efficacy of TMS techniques. Secondary outcomes include changes in symptoms measured by the HAM-D17 score, treatment response defined as a ≥ 50% reduction in HAM-D17 score, changes in quality of life assessed with the WHOQOL-BREF, and treatment safety and tolerability.

**Table 2:** Summary of study results

Authors	Treatment	Study Design	Limitations
(Wirecki et al. 2015)	Stimulation was administered over a similar period (4 weeks, 5 sessions per week), totaling 20 sessions, with the H1 coil applied for deep stimulation and the figure-8 coil for superficial stimulation. The protocols followed FDA approvals, aiming to test differences in stimulation depth and amplitude compared to previous studies, with a focus on symptom remission and treatment safety. Efficacy was measured through changes in the Beck Depression Inventory score. Each group received TMS treatment five days per week, totaling 20 sessions. Randomization was conducted with stratified blocks, ensuring balanced distribution of age and gender across groups	This observational study evaluated patients in a real-world clinical setting, comparing treatment response between rTMS (figure-8 coil) and dTMS (H1 coil) devices, with a particular focus on the population with MDD.	Small sample size and unbalanced distribution: The study included only 66 patients, with 45 in the rTMS group and 21 in the dTMS group. This difference in sample size between groups reduces statistical power and may affect the reliability of the results, limiting the ability to generalize the conclusions to a broader population. Lack of long-term follow-up: The study focused on the effects of TMS after 20 sessions, without examining long-term outcomes or the maintenance of symptom remission. This limits understanding of the duration of effectiveness for both treatments. Absence of an untreated control group: Despite positive results for both treatments, the lack of an untreated or placebo-only control group limits the ability to compare the efficacy of TMS against observation alone or other inactive treatment modalities. Single-center: Conducted in a single clinical center, the results may not be representative of a broader population or diverse clinical settings. Multicenter studies would be needed to strengthen the generalizability of the findings.
(Filipčić et al. 2018)	This study was a randomized controlled trial comparing the efficacy and safety of TMS using the H1 (deep) coil versus the figure-8 coil. Stimulation was applied five days per week for four consecutive weeks. The H1 coil protocol was set to stimulate deeper brain regions, while the figure-8 coil specifically targeted the left dorsolateral prefrontal cortex (DLPFC). The protocols used followed FDA-approved guidelines for treatments with the H1 and figure-8 coils in treating TRD. dTMS with H1 coil: 20-minute sessions at 18 Hz, at 120% of motor threshold, with 55 pulse trains totaling 1980 pulses per session. rTMS with figure-8 coil: 40-minute sessions at 10 Hz, at 120% of motor threshold, with 75 pulse trains totaling 3000 pulses per session.	Single-center, single-blind, randomized controlled clinical trial conducted in Croatia. Study arms: (1) rTMS with H1 coil, (2) rTMS with figure-8 coil, (3) standard therapy only. Participants were randomly assigned to the three groups in a 1:1:1 ratio using stratified block randomization, with stratification by age and gender.	Sample Size: Although the study aims to enroll 76 patients in each group, the overall sample size may still be considered small for generalizing the findings to a broader population. A larger sample size could provide more robust data and enhance the statistical power of the results. Single-Center Design: The study is conducted at a single center, which may limit the generalizability of the findings. Results may vary in different clinical settings or populations, and multicenter trials could provide a more comprehensive understanding of the treatment’s efficacy and safety. Short Duration of Follow-Up: The primary outcomes are assessed at the end of week 4, which may not be sufficient to evaluate the long-term effects of rTMS. Longer follow-up periods are necessary to determine the sustainability of treatment effects and any potential late-onset side effects. Single-Blinded Design: The study is single-blinded, meaning that only the participants are unaware of the treatment they receive. This design may introduce bias, as the researchers conducting assessments may inadvertently influence the results based on their knowledge of the treatment allocation. Exclusion Criteria: The strict exclusion criteria may limit the applicability of the findings to a wider range of patients with MDD. For instance, individuals with significant comorbidities or those who have previously undergone rTMS are excluded, which may not reflect the real-world clinical population. Subjective Measures: The study relies on self-reported measures and clinician-administered scales to assess outcomes. This reliance on subjective assessments may introduce bias and variability in the results, as patients’ perceptions of their symptoms can be influenced by various factors. Lack of Control for Confounding Variables: While the study aims to control for certain variables, there may still be unmeasured confounding factors that could influence the outcomes, such as lifestyle factors, Variability in rTMS Protocols: Differences in rTMS protocols, such as frequency, intensity, and duration of treatment, may affect the outcomes. Standardizing these parameters across studies is essential for comparing results and drawing definitive conclusions.
(Filipčić et al. 2019)	Similar to the previous 2018 study, this study examined the efficacy and safety of two rTMS modalities (H1 coil and figure-8 coil) in patients with TRD. Patients received 20 rTMS sessions over 4 weeks, with five sessions per week, using either the H1 coil (18 Hz, 1980 pulses) or the figure-8 coil (10 Hz, 3000 pulses), in addition to standard pharmacotherapy, or standard pharmacotherapy alone. As in the prior study, the H1 coil targeted deeper brain areas, while the figure-8 coil stimulated superficial cortical areas. Monitoring included assessments of quality of life and symptom response through standardized scales such as the WHOQOL-BREF and HAM-D17 scores.	Pragmatic, randomized, single-blind, controlled study evaluating the efficacy and safety of high-frequency rTMS (HF-rTMS) using the H1 coil compared to the figure-8 coil, as an adjunct to standard pharmacotherapy in patients with TRD.	Control group monitoring: Patients treated with rTMS modalities were monitored daily, while the control group was only assessed at baseline and after 4 weeks of treatment. This difference in monitoring frequency may have introduced bias against the null hypothesis, potentially overestimating the effectiveness of the two rTMS modalities. Lack of a sham control group: A sham device was not used to control for both active modalities, which would have allowed for a more rigorous comparison. Absence of long-term assessments: The study did not include long-term outcome evaluations or assessments of neurocognitive functions, limiting understanding of the treatment’s lasting effects. Single-center study: Conducted in a single center, which may reduce the generalizability of results to a broader population. Non-uniform treatment protocols: The treatment parameters for the two TMS modalities were not standardized; pulse frequency, total pulses per session, and train duration differed between modalities. The study aimed to evaluate FDA-approved rTMS protocols commonly used in clinical practice. Researchers sought to reflect real-world practices where protocols vary depending on factors like clinician preferences or patient characteristics. However, this lack of uniformity in treatment protocols can make it more challenging to directly compare the efficacy of the two modalities.

## Results

The three studies under review report findings that demonstrate the efficacy of both rTMS and dTMS for the treatment of MDD, although some differences are noted in the outcomes and measures employed.

- 2015 Study: This observational study showed significant improvements in Beck Depression Inventory scores for both the rTMS and dTMS groups, with a similar average reduction in depressive symptoms. However, the results did not show a significant difference between the two groups, suggesting that both rTMS and dTMS are effective but not necessarily superior to one another in a real-world clinical setting. The treatment response rate was high in both groups, though the study carries an overall risk of bias, mainly due to its observational nature and lack of randomization. In the article by Wirecki et al. 2015; no further data is provided, whereas for the two trials, a detailed explanation of the figure-8-coil and H1-coil protocols used for transcranial magnetic stimulation is included:

1. **Figure-8-coil**:

- Session Duration: 40 min.
- Number of Pulses: Delivered at 10 Hz (4-second trains separated by 26-second inter-train intervals, totaling 75 trains and 3000 pulses per session), higher than the H1-coil as the standard figure-8-coil protocol involves a greater number of pulses per session with a stimulation intensity of 120% of the motor threshold.
- Stimulation Target: The figure-8 coil, previously used in studies leading to its FDA approval, stimulates the left dorsolateral prefrontal cortex (L-DLPFC).

2. **H1-coil**:

- Session Duration: 20 min.
- Number of Pulses: Delivered at 18 Hz (2-second trains separated by 20-second inter-train intervals, totaling 55 trains and 1980 pulses per session). The standard H1-coil protocol uses fewer pulses than the figure-8-coil.
- Stimulation Target: The H1-coil is designed to stimulate broader and deeper volumes in the prefrontal cortex, potentially increasing the likelihood of targeting projections that affect the subgenual anterior cingulate cortex.
- 2018 RCT Study: In this randomized RCT, the results showed that both the H1-coil (dTMS) and figure-8 coil (rTMS) were effective in reducing depressive symptoms. The outcome focused on assessing the short-term efficacy and tolerability of high-frequency repetitive transcranial magnetic stimulation compared to deep transcranial therapy in major depressive disorder. Both treatments demonstrated good safety and tolerability, with few significant adverse effects reported. The study highlights the need for further research to deepen understanding of TMS mechanisms of action, long-term efficacy, and its impact on specific symptoms and comorbid conditions associated with MDD. Among the exploratory objectives were the analysis of symptoms such as hope and insomnia, as well as the assessment of biomarkers like baseline plasma BDNF levels, which have potential predictive value for treatment outcomes. The correlation between BDNF levels and treatment results could offer valuable insights for personalizing therapies and advancing the understanding of depression. The study’s conclusions support the integration of TMS in treatment protocols for MDD, encouraging clinicians to consider this modality as part of a multimodal approach that includes pharmacotherapy and psychotherapy.
- 2019 RCT Study: This randomized controlled trial evaluated the efficacy of TMS using the H1-coil and the figure-8 coil in 228 patients with treatment-resistant major depressive disorder (TRD), divided into three groups: H1-coil, figure-8 coil, and a control group receiving only standard pharmacotherapy. At the end of the treatment period (20 sessions), remission (defined as HAM-D17 ≤ 7) was achieved by 60% of patients in the H1-coil group, 43% in the figure-8 coil group, and 11% in the control group.

Although remission rates did not differ significantly between the two active treatment groups, the treatment response rate (defined as ≥ 50% reduction in HAM-D17 score) was significantly higher in the H1-coil group, with an odds ratio (OR) of 2.33 compared to the figure-8 group. Similarly, the mean reduction in HAM-D17 score was significantly greater with the H1 coil (59%) compared to the figure-8 coil (41%) and the control group (17%), with statistical significance for the difference between the two TMS modalities (*P* = 0.048).

Despite the marked reduction in depressive symptoms, improvements in quality of life did not differ significantly between the three groups, suggesting a possible dissociation between symptomatic remission and perceived well-being. All treatment arms demonstrated good tolerability, with similar dropout rates (9.7% H1-coil, 4% figure-8 coil, 11.1% control). The study concludes that both TMS treatments are effective and safe for treatment-resistant major depressive disorder, with the H1 coil demonstrating greater efficacy in reducing symptoms. However, given the lack of double-blind studies, multicenter, long-term studies may be needed to confirm these findings and further investigate the impact on quality of life.

Both protocols were administered as adjunct treatments to standard pharmacotherapy for MDD. Patients were randomized to receive 20 sessions of TMS with one of the two devices or standard pharmacotherapy alone. The studies followed an FDA-approved protocol commonly used for both devices, ensuring that procedures adhered to clinical standards. In the latest study, several side effects associated with the two repetitive transcranial magnetic stimulation (rTMS) protocols, namely the H1-coil and figure-8-coil, were also reported. Here is a summary of the side effects observed:

1. **Figure-8-coil**:

- Headache: 15 patients (20%).
- Discomfort at application site: 1 patient (1%).
- Dizziness or light vertigo: 2 patients (3%).
- Anxiety: 1 patient (1%).
- Insomnia: 5 patients (7%).

2. **H1-coil**:

- Headache: 20 patients (29%).
- Discomfort at application site: 3 patients (4%).
- Pain at application site: 5 patients (7%).
- Muscle twitching/spasms or jaw pain: 8 patients (12%).
- Dizziness or light vertigo: 4 patients (6%).
- Insomnia: 5 patients (7%).

3. **Control Group (pharmacotherapy only)**:

- Headache: 3 patients (4%).
- Dizziness: 1 patient (1%).
- Anxiety: 2 patients (3%).
- Fatigue: 2 patients (3%).
- Nausea: 1 patient (1%).
- Insomnia: 4 patients (5%).

Overall, both TMS protocols were well-tolerated, with mild side effects and no serious adverse events reported. Headaches were the most common side effect in both TMS groups.

**Fig. 2:**
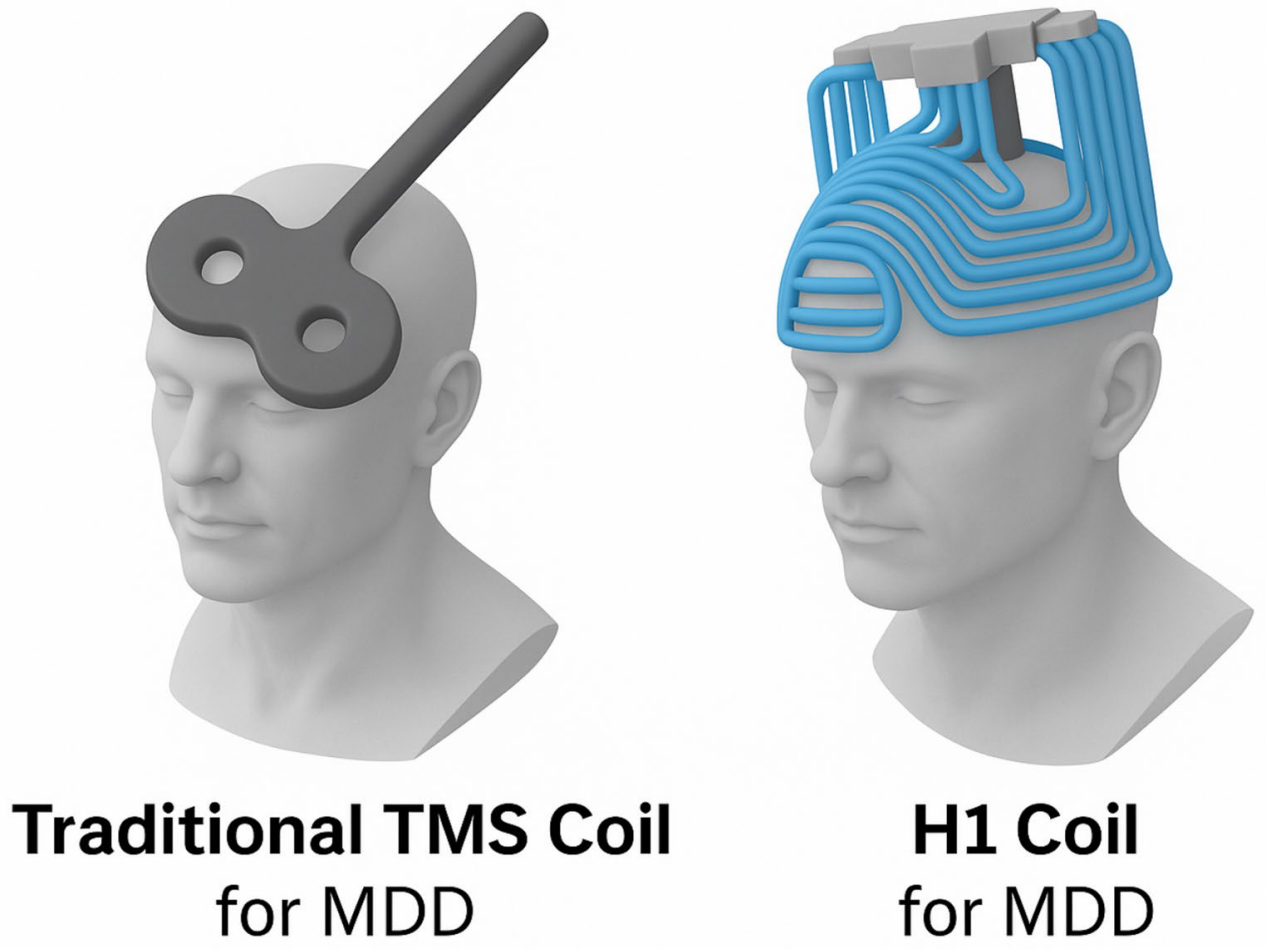
Different coils

Although protocol differences are frequently reported in studies comparing rTMS and dTMS, these variations do not imply methodological inconsistencies or flaws in study design. Rather, they reflect the intrinsic technical specifications, engineering constraints, and safety regulations associated with each device. Key differences in coil geometry, energy delivery, and field distribution play a central role in shaping the stimulation protocols required for each modality. For example, the traditional figure-of-8 coil used in rTMS generates focal magnetic fields with a penetration depth of approximately 1.5 to 2.5 cm. Due to its design, energy dissipates rapidly at the edges of the coil, which necessitates longer inter-train intervals to manage thermal load and avoid overheating. However, the introduction of cooling systems in newer figure-of-8 coil models has mitigated this limitation, allowing for the safe administration of higher frequency protocols—up to 18 Hz—that were previously restricted to dTMS systems (Rossi et al. 2021);(Ueno and Sekino 2021). Additionally, the effectiveness of rTMS is highly dependent on accurate coil positioning over the left dorsolateral prefrontal cortex (DLPFC), since the magnetic field diminishes proportionally to the square of the distance from the coil (Barker et al. 1985). In contrast, dTMS employs H-coils, such as the H1 coil, which are designed with a multi-wing configuration and a three-dimensional winding pattern (see Fig. 2). This structure enables stimulation to reach deeper brain regions—approximately 4 to 5 cm below the skull—while simultaneously activating broader neural circuits (Levkovitz et al. 2015). The distributed nature of the coil windings allows for more efficient heat dispersion, which in turn permits shorter inter-train intervals even when higher energy output is applied (Rastogi et al. 2019). The increased depth of stimulation, however, comes at the expense of spatial precision. While the figure-of-8 coil can selectively target cortical areas such as the DLPFC, the H1 coil reaches subcortical structures, including the anterior cingulate cortex, with less focal accuracy—a trade-off that may be clinically advantageous in certain phenotypes of depression. Protocol parameters, such as pulse width and current direction, are also adapted to the coil design. rTMS protocols typically use narrower pulses (e.g., 100 µs) and anteroposterior current orientation to optimize superficial cortical activation. Conversely, dTMS uses wider pulses (e.g., 300 µs), which facilitate deeper tissue penetration, albeit at the cost of requiring lower stimulation frequencies (e.g., 10 Hz compared to the 18 Hz used in modern rTMS systems) to prevent excessive energy deposition and ensure patient safety (Lefaucheur et al. 2020). Both systems operate under strict regulatory frameworks defined by FDA and CE guidelines. rTMS protocols generally involve a higher number of pulses per session (e.g., 3,000 pulses at 10 Hz) due to the narrower area of stimulation, while dTMS sessions include fewer pulses (e.g., 1,980 at 18 Hz) because each pulse covers a broader neural volume, thus reducing seizure risk (Rossi et al. 2021). Treatment schedules also differ: dTMS protocols typically emphasize condensed, intermittent courses—20 sessions over 4 weeks—balancing efficacy with tolerability (Rapinesi et al. 2015), whereas rTMS often requires more prolonged interventions, such as 30 to 40 sessions, to achieve comparable results. In this light, the observed variability in stimulation protocols between rTMS and dTMS should not be interpreted as a methodological weakness, but rather as a reflection of the technical optimization strategies and distinct clinical objectives inherent to each neuromodulation system.

## Discussion

This systematic review highlights key observations regarding the use of rTMS and deep dTMS in the treatment of treatment-resistant depression (TRD). Although dTMS has received approval from the U.S. Food and Drug Administration (FDA) for TRD, clinical studies directly comparing its efficacy with that of rTMS remain limited. The scarcity of such comparative research, as evidenced in our review, is particularly noteworthy and warrants thorough examination. Understanding the underlying reasons for this paucity is not straightforward. One possible explanation is that, for over a decade, the FDA has granted dTMS a regulatory status equivalent to that of rTMS for TRD, implicitly recognizing their comparable efficacy. This equivalence may have reduced the perceived need for head-to-head clinical comparisons, despite the intrinsic differences in physical mechanisms and engineering design between the two stimulation systems. Another plausible factor is the high cost associated with real-world studies involving dTMS. These devices require sophisticated and expensive technology, alongside specific and often complex maintenance procedures. Such financial and logistical barriers may limit the feasibility of large, multicenter, randomized clinical trials and consequently hinder the production of robust comparative evidence. In this context, systematic reviews and meta-analyses may offer a viable methodological alternative. While they cannot fully substitute for primary clinical trials, they allow for the integration and synthesis of existing data, thereby providing valuable and accessible insights at a lower economic and organizational cost. This approach may help to inform clinical decision-making while overcoming some of the practical constraints associated with experimental studies. Indeed, our review identified only three comparative studies: one with limited clinical value, and two others characterized by methodological transparency and well-defined primary and secondary outcomes. The shortage of direct comparisons between rTMS and dTMS constitutes a critical gap in the literature, particularly given the importance of evidence-based guidelines to inform clinical practice. At present, two ongoing double-blind randomized trials in France and Canada are expected to contribute further high-quality data in the near future. Their results could help bridge the existing knowledge gaps and provide a more precise assessment of the comparative efficacy and cost-effectiveness of rTMS and dTMS—two factors that are especially relevant in resource-constrained healthcare settings.

## Conclusions

The three studies we identified confirm that both rTMS with figure-8 coil and dTMS with H1 coil are effective in reducing depressive symptoms, without significant differences in remission rates. Both treatments are well-tolerated, with minimal and manageable side effects, though dTMS, due to the deeper stimulation, may cause slightly more discomfort. However, dTMS incurs higher costs due to the advanced technology required, making it less sustainable in settings with limited budgets, where rTMS might represent a more practical option. Although not all studies explicitly address costs, it is evident that dTMS is more costly due to the advanced technology, such as the H1 coil, and associated maintenance expenses. More affordable, rTMS appears preferable in budget-limited contexts.

In summary, the results indicate that both rTMS and dTMS offer clinical efficacy and a favorable safety profile for the management of treatment-resistant depression. Although no significant differences in symptomatic remission were observed, dTMS may offer a slight advantage in complex clinical settings, such as cases of TRD accompanied by psychiatric or neurological comorbidities, childhood trauma, cognitive impairment, a history of multiple pharmacological treatment failures, or poor adherence. These cases often align with the broader and clinically nuanced category of difficult-to-treat depression, which encompasses not only TRD but also patients with partial responses, fluctuating adherence, and concomitant clinical complications that hinder standard treatment. Further randomized, multicenter trials with extended follow-up are warranted to validate these findings, investigate patient-reported outcomes such as quality of life, and deepen our understanding of the underlying neurobiological mechanisms. Although dTMS devices entail higher initial and maintenance costs, the clinical benefits of the H1 coil for patients with complex symptoms may justify its use. As neurostimulation technologies advance, TMS protocols could become increasingly personalized, tailored to individual patient characteristics. Robust evidence from future studies will be essential to achieve truly personalized neuromodulation in psychiatry.

## Electronic Supplementary Material

Below is the link to the electronic supplementary material.

Supplementary Material 1 Figure 2
