# Supplementary figures and images for: Comparison of therapeutic efficacy in depression between repetitive TMS and deep TMS
Source: J Neural Transm (Vienna). 2025 May 27;132(8):1113–24. doi: 10.1007/s00702-025-02944-w (PMC12479554; doi:10.1007/s00702-025-02944-w)

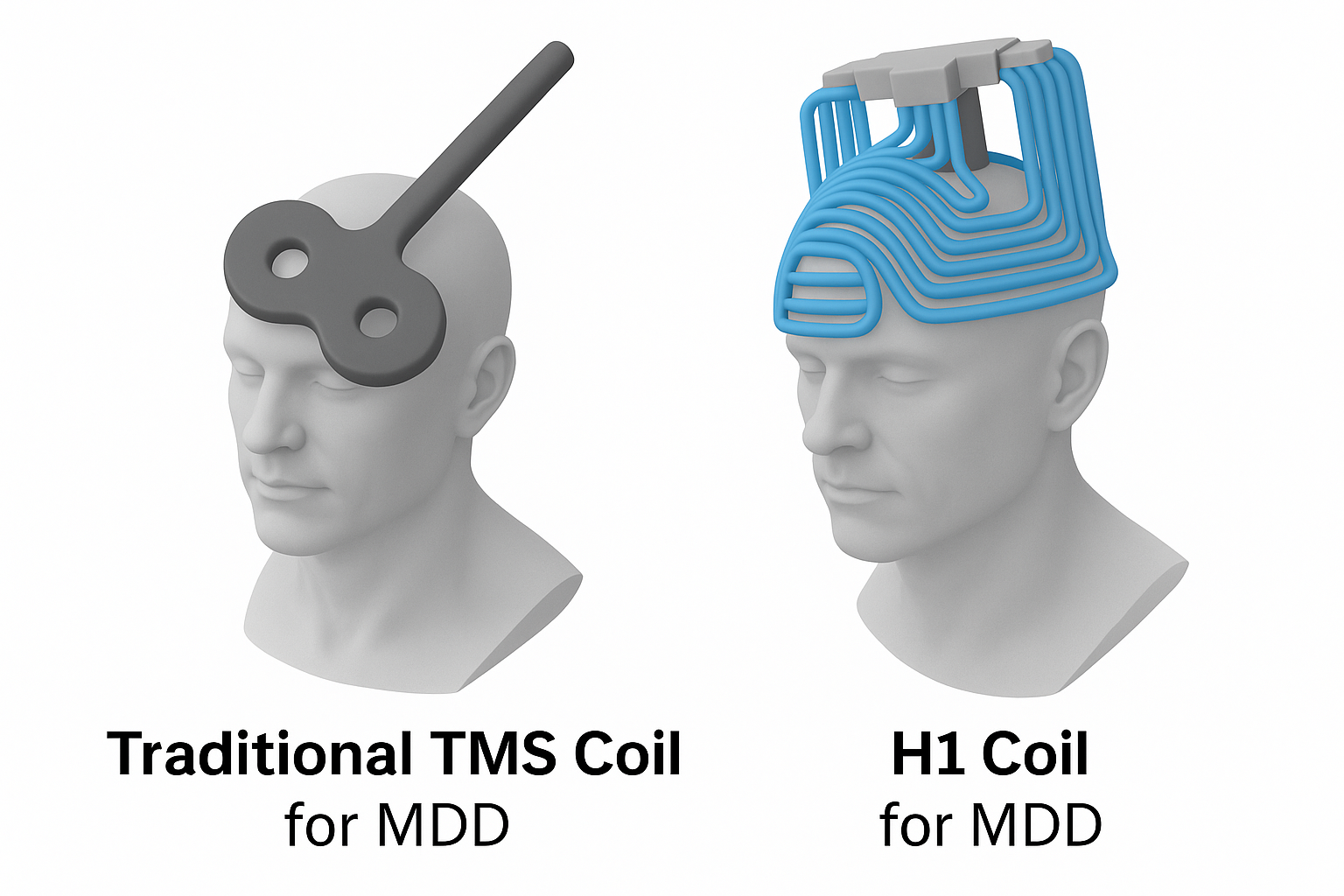

Supplement: Supplementary file 1 — Supplementary Material 1 Figure 2 [file 702_2025_2944_MOESM1_ESM.png]
